# The CXCL12 G801A Polymorphism Is Associated with Cancer Risk: A Meta-Analysis

**DOI:** 10.1371/journal.pone.0108953

**Published:** 2014-09-30

**Authors:** Ke Zhu, Benchun Jiang, Rong Hu, Ying Yang, Miao Miao, Yingchun Li, Zhuogang Liu

**Affiliations:** 1 Department of Hematology, Affiliated Shengjing Hospital, China Medical University, Shenyang, Liaoning, China; 2 Department of General Surgery, Affiliated Shengjing Hospital, China Medical University, Shenyang, Liaoning, China; University of Birmingham, United Kingdom

## Abstract

**Background:**

CXCL12 is a small chemotactic cytokine belonging to the CXC chemokine family expressed in various organs. It contributes to the migration, invasion and angiogenesis of cancer cells. Recently, the CXCL12 G801A polymorphism was shown to be associated with an increased risk of various kinds of cancers, but the results were too inconsistent to be conclusive.

**Methods:**

To solve the problem of inadequate statistical power and conflicting results, a meta-analysis of published case-control studies was performed, including 4,435 cancer cases and 6,898 controls. Odds ratios (ORs) and their 95% confidence intervals (CIs) were used to determine the strength of association between CXCL12 G801A polymorphism and cancer risk.

**Results:**

A significant association between CXCL12 G801A polymorphism and cancer risk was found under all genetic models. Further, subgroup analysis stratified by ethnicity suggested a significant association between CXCL12 G801A polymorphism and cancer risk in the Asian subgroup under all genetic models. However, in the Caucasian subgroup, a significant association was only found under an additive genetic model and a dominant genetic model. The analysis stratified by cancer type found that CXCL12 G801A polymorphism may increase the risk of breast cancer, lung cancer, and “other” cancers. Based on subgroup stratified by source of controls, a significant association was observed in hospital-based studies under all genetic models.

**Conclusions:**

The CXCL12 G801A polymorphism is associated with an increased risk of cancer based on current published data. In the future, large-scale well-designed studies with more information are needed to better estimate possible gene-gene or gene-environment interactions.

## Introduction

Chemokines are small glycoproteins that contribute to the regulation of various biological processes [1]. CXCL12, also known as stromal cell-derived factor 1(SDF-1), is a small chemotactic cytokine belonging to the CXC chemokine family that is constitutively expressed in various organs [2]. It contributes to the regulation of leukocyte trafficking and many essential biological processes, including cardiac and neuronal development, stem cell motility, neovascularization, and tumorigenesis [3]–[7].

CXCL12 binds primarily to the CXCR4 receptor, resulting in a CXCL12/CXCR4 receptor-ligand system involving a one-on-one interaction [8], [9]. CXCR4 may play a vital role in the metastatic processes of many types of cancers, including colorectal, breast and oral squamous cell carcinoma [10]–[12]. Further research has emphasized the key role of CXCR4 in tumor cell malignancy; the activation of CXCR4 by CXCL12 has been shown to induce the migration, invasion and angiogenesis of tumor cells [13], [14].

CXCL12 is located on chromosome 10q11.1 and has a G→A mutation at position 801 in the 3′-untranslated region in its β transcriptional splice variant [15], [16]. The CXCL12 G801A polymorphism may be essential to increasing the production of a CXCL12 protein that has been shown to be associated with an increased risk of various kinds of cancers, such as breast cancer, lung cancer and lymphoma [17]–[19]. Recently, numerous studies have shown that the CXCL12 G801A polymorphism occurs in different types of cancers, but the results have been too inconsistent to be conclusive. In addition, the sample size of each study is relatively small; thus, their statistical power is too low to detect associations between the CXCL12 G801A polymorphism and cancer risk. Meta-analysis is a powerful method for resolving inconsistent findings from a relatively large number of subjects. To solve the problem of inadequate statistical power and conflicting results, we performed this meta-analysis of published case-control studies.

## Materials and Methods

### Literature Search

Two investigators independently searched for eligible studies of the associations between CXCL12 G801A polymorphism and cancer risk. Studies published through March 2014 were identified through a computerized search of PubMed without language limitation. The key words used in this search were as follows: (CXCL12, SDF-1 or rs1801157) and (cancer, tumor, carcinoma or neoplasm) and polymorphism. The references of all identified publications were also searched for additional studies. Studies included in this meta-analysis had to meet the following inclusion criteria: (a) used a case-control study design, (b) evaluated CXCL12 G801A polymorphism and cancer risk, (c) reported detailed genotype frequencies of cases and controls or these could be calculated from the text of the manuscript, and (d) the control subjects were in agreement with the Hardy-Weinberg equilibrium (HWE).

### Data Extraction

Two investigators extracted the data independently, and disagreements were settled by discussion. The following data were extracted from the eligible studies: the first author's name, year of publication, country of origin, ethnicity, the source of controls, and numbers of genotyped cases and controls. If the data was not available, study authors were contacted to request missing data.

### Statistical Analysis

ORs and their 95% CIs were used to determine the strength of association between the CXCL12 G801A polymorphism and cancer risk. The significance of the pooled OR was determined using the Z test, and *P*<0.05 was considered statistically significant. Additive (A vs. G), dominant (GA+AA vs. GG), and recessive (AA vs. GG+GA) genetic models were investigated. Subgroup analysis was performed by ethnicity, cancer type (if one cancer type contained less than two studies, it was defined as “other”), and source of controls, either hospital or population controls. HWE was tested using the chi-square test among controls, and *P*<0.05 was considered a significant departure from HWE. If the *P* value for heterogeneity was >0.05 and *I* ^2^<50%, indicating an absence of heterogeneity between studies, the fixed-effects model (the Mantel-Haenszel method) was used. In contrast, if either the *P* value for heterogeneity was ≤0.05 or *I* ^2^ was ≥50%, indicating heterogeneity among the studies, the more appropriate random-effects model (the DerSimonian and Laird method) was used. Sensitivity analyses were performed to assess the stability of the results. Funnel plots and Egger's linear regression test were used to diagnose potential publication bias, and *P*<0.05 was used to indicate possible publication bias. All analyses were performed using Stata software. *P* values were based on two-sided tests.

## Results

### Characteristics of Eligible Studies

**Figure 1:**
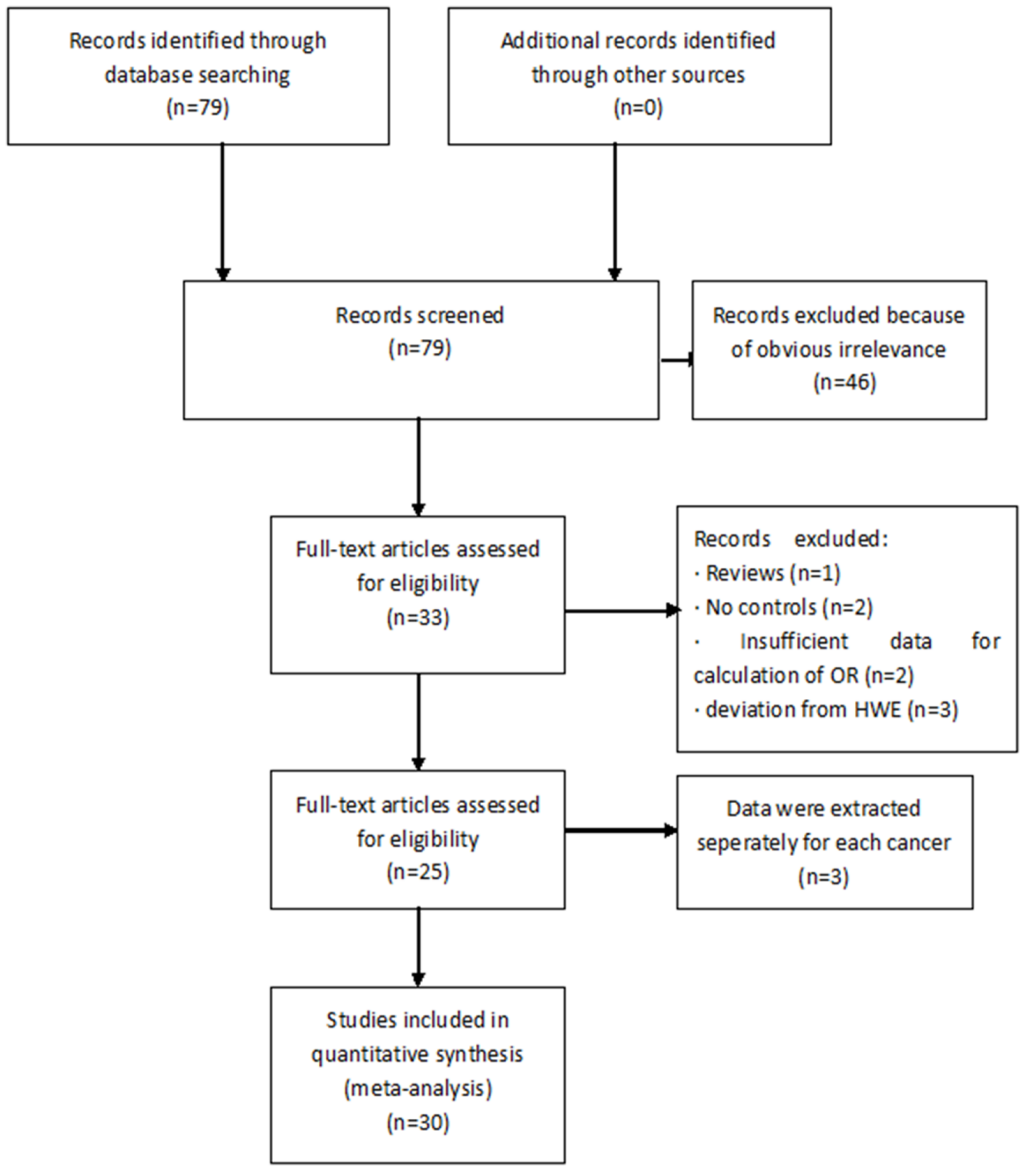
Flow chart of study selection in the meta-analysis.

Our meta-analysis was performed according to guidelines of the “Preferred Reporting Items for Systematic Reviews and Meta-Analyses” (PRISMA) statement (Checklist S1) and “Meta-analysis on Genetic Association Studies” statement (Checklist S2). Figure 1 graphically illustrates the study flow chart. The literature search yielded 79 potentially relevant articles. After screening the titles and abstracts, 46 articles were excluded because of obvious irrelevance. In addition, after reading the full text of the 33 remaining articles, 8 articles were excluded for the following reasons: article was a review (n = 1), articles lacked controls (n = 2), articles had insufficient data (n = 2), and articles deviated from HWE (n = 3). Articles that reported data for different types of cancers were treated as independent studies. Thus, 25 articles [17]–[41] (30 independent case-control studies) met the inclusion criteria; they included 4,435 cancer cases and 6,898 controls. Data collected from the included studies are summarized in Table 1.

**Table 1:** Characteristics of eligible studies included in the meta-analysis.

*author*	*year*	*cancer*	*country*	*ethnicity*	*control source*	*HWE*	*Cases*			*controls*		
							GG	GA	AA	GG	GA	AA
Zafiropoulos [17]	2004	breast cancer	Greece	Caucasian	HB	0.764	98	136	30	101	92	19
	2004	bladder cancer	Greece	Caucasian	HB	0.124	31	32	5	67	71	10
	2004	skin cancer	Greece	Caucasian	HB	0.262	64	38	8	169	164	30
Razmkhah [18]	2005	lung cancer	Iran	Asian	HB	0.504	25	38	9	145	97	20
Razmkhah [20]	2005	breast cancer	Iran	Asian	HB	0.682	105	139	34	101	67	13
Hidalgo-Pascual [21]	2007	colorectal cancer	Spain	Caucasian	PB	0.77	212	128	9	319	172	25
Hirata [22]	2007	prostate cancer	Japan	Asian	HB	0.651	72	78	17	91	63	13
Dimberg [23]	2007	colorectal cancer	Sweden	Caucasian	HB	0.117	84	62	5	81	56	4
de Oliveira [24]	2007	CML	Brazil	Caucasian	HB	0.628	10	11	4	39	18	3
Khademi [25]	2008	head and neck cancer	Iran	Asian	HB	0.504	64	84	8	145	97	20
Vairaktaris [26]	2008	oral cancer	Mixed	Caucasian	PB	0.448	104	51	4	55	41	5
Lin [27]	2009	breast cancer	China	Asian	HB	0.621	106	98	16	175	136	23
Vazquez-Lavista [28]	2009	bladder cancer	Mexico	Mixed	PB	0.822	29	15	3	83	39	4
de Oliveira [19]	2009	breast cancer	Brazil	Caucasian	HB	0.939	59	41	3	61	32	4
	2009	NHL	Brazil	Caucasian	HB	0.356	36	33	1	59	26	5
	2009	HL	Brazil	Caucasian	HB	0.356	22	10	4	59	26	5
Kruszyna [29]	2010	breast cancer	Poland	Caucasian	PB	0.686	123	61	9	136	58	5
Kruszyna [30]	2010	laryngeal cancer	Poland	Caucasian	PB	0.114	69	46	3	181	67	2
Lee [31]	2011	NSCLC	China	Asian	HB	0.379	99	112	36	171	136	21
de Oliveira [32]	2011	breast cancer	Brazil	Caucasian	HB	0.758	32	21	2	37	15	2
Cacina [33]	2012	endometrial cancer	Turkey	Asian	HB	0.061	49	52	12	69	64	6
Tee [34]	2012	cervical cancer	Taiwan	Asian	HB	0.697	37	29	10	164	140	33
Kucukgergin [35]	2012	bladder cancer	Turkey	Asian	HB	0.35	58	58	26	94	80	23
Liarmakopoulos [36]	2013	gastric cancer	Greece	Caucasian	HB	0.116	39	43	6	205	229	46
Perim [37]	2013	ALL	Brazil	Caucasian	PB	0.72	33	18	3	46	11	1
Razmkhah [38]	2013	gastric cancer	Iran	Asian	HB	0.504	66	48	10	145	97	20
	2013	colorectal cancer	Iran	Asian	HB	0.504	62	39	8	145	97	20
Shi [39]	2013	colorectal cancer	Taiwan	Asian	PB	0.1	141	113	4	248	52	0
Kontogianni [40]	2013	breast cancer	Greece	Caucasian	HB	0.585	114	118	29	247	198	35
Cai [41]	2013	renal cell cancer	China	Asian	HB	0.127	150	111	61	237	136	29

CML: chronic myeloid leukemia; NHL: non-hodgkin lymphoma; HL: hodgkin lymphoma; NSCLC: non small cell lung cancer; ALL:acute lymphocytic leukemia; HB: hospital-based; PB:population-based.

### Results of the Meta-analysis

**Figure 2:**
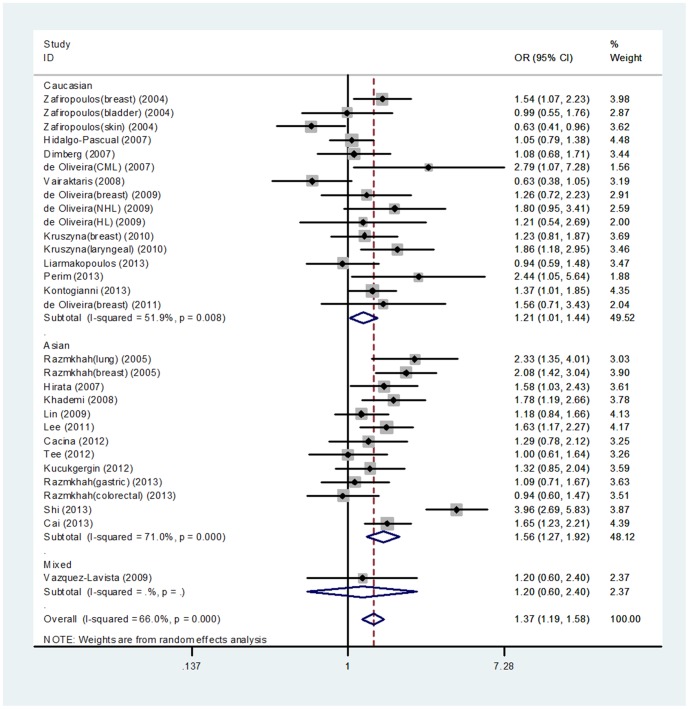
Forest plot of CXCL12 G801A polymorphism and cancer risk under a dominant genetic model (GA+AA vs. GG) stratified by ethnicity.

A significant association between CXCL12 G801A polymorphism and cancer risk was found under an additive genetic model (OR = 1.30, 95% CI = 1.16–1.45), a dominant genetic model (OR = 1.37, 95%CI = 1.19–1.58), and a recessive genetic model (OR = 1.38, 95% CI = 1.13–1.69). Subgroup analysis stratified by ethnicity also suggested a significant association between CXCL12 G801A polymorphism and cancer risk in the Asian subgroup under an additive genetic model (OR = 1.45, 95% CI = 1.23–1.70), a dominant genetic model (OR = 1.56, 95%CI = 1.27–1.92) (Figure 2), and a recessive genetic model (OR = 1.71, 95% CI = 1.41–2.07). In the Caucasian subgroup, a significant association was found under an additive genetic model (OR = 1.16, 95%CI = 1.00–1.34) and a dominant genetic model (OR = 1.21, 95%CI = 1.01–1.44).

Furthermore, in the analysis by stratified cancer type, a significantly increased risk was found in breast cancer and lung cancer under all genetic models. In addition, under the additive and dominant genetic models, a significantly increased risk was found in “other” cancers. However, no significant association with this polymorphism was observed in bladder, colorectal and gastric cancers. Base on subgroup analysis by source of controls (hospital or population controls), a significant association was observed in hospital-based studies under all genetic models (Table 2).

**Table 2:** Pooled ORs and 95% CIs of the association between CXCL12 G801A polymorphism and cancer risk.

	*A vs G*			*GA/AA vs GG*			*AA vs GA/GG*		
	OR(95%CI)	*I* ^2^(%)	P-value	OR(95%CI)	*I* ^2^(%)	P-value	OR(95%CI)	*I* ^2^(%)	P-value
overall	1.30(1.16–1.45)	67.3	0.000	1.37(1.19–1.58)	66.0	0.000	1.38(1.13–1.69)	33.3	0.041
ethnicity									
Asian	1.45(1.23–1.70)	70.8	0.000	1.56(1.27–1.92)	71.0	0.000	1.71(1.41–2.07)	40.8	0.062
Caucasian	1.16(1.00–1.34)	52.9	0.007	1.21(1.01–1.44)	51.9	0.008	1.11(0.87–1.41)	12.0	0.316
Cancer type									
Breast cancer	1.32(1.17–1.48)	0.0	0.537	1.43(1.23–1.66)	0.0	0.436	1.41(1.06–1.87)	0.0	0.843
Bladder cancer	1.22(0.97–1.55)	0.0	0.577	1.19(0.87–1.62)	0.0	0.731	1.58(0.96–2.61)	0.0	0.744
Lung cancer	1.65(1.34–2.04)	0.0	0.610	1.80(1.36–2.39)	17.6	0.271	2.24(1.41–3.57)	0.0	0.475
colorectal cancer	1.33(0.76–2.34)	91.6	0.000	1.43(0.73–2.80)	91.7	0.000	0.86(0.53–1.41)	36.6	0.192
Gastric cancer	0.98(0.77–1.25)	0.0	0.505	1.02(0.74–1.39)	0.0	0.637	0.87(0.48–1.55)	0.0	0.476
Others	1.31(1.06–1.61)	68.8	0.000	1.36(1.05–1.75)	66.7	0.001	1.47(0.96–2.26)	51.3	0.020
Source of controls									
HB	1.27(1.15–1.41)	49.1	0.004	1.34(1.18–1.52)	45.0	0.011	1.49(1.28–1.75)	28.1	0.105
PB	1.42(0.94–2.14)	86.8	0.000	1.49(0.93–2.40)	86.6	0.000	1.09(0.69–1.73)	46.1	0.085

### Sensitivity Analysis

A single study was excluded each time to evaluate the effect of an individual study on the combined ORs and 95% CIs. The omission of any single study did not significantly change the pooled effects of the additive, dominant and recessive genetic models; these findings confirmed that the meta-analysis results were statistically robust and that our results were reliable and stable (data not shown).

### Publication Bias

**Figure 3:**
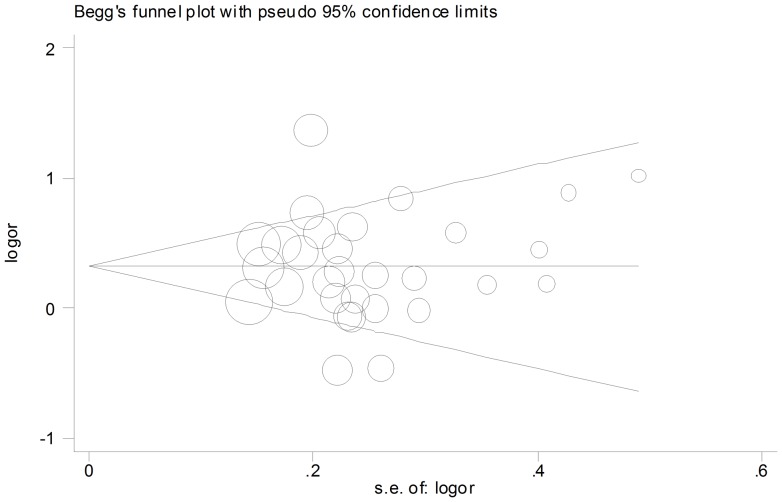
Funnel plot for studies of the association of CXCL12 G801A polymorphism and cancer risk under a dominant genetic model (GA+AA vs. GG).

Begg's funnel plot and Egger's test were performed to assess the publication bias of this set of publications. The shape of the funnel plot did not show obvious publication bias (Figure 3). Similarly, Egger's test revealed no evidence of publication bias (P = 0.996 for the additive genetic model; P = 0.953 for the dominant genetic model; and P = 0.342 for the recessive genetic model).

## Discussion

CXCL12 is primarily produced by stromal cells and is important for the growth, angiogenesis and metastasis of tumor cells [42], [43]. The CXCL12 G801A polymorphism may be essential to increasing the production of CXCL12 protein. Furthermore, overexpression of CXCL12 is associated with the development and metastasis of many kinds of cancers. The CXCL12 G801A polymorphism has been investigated in various types of cancers. However, the results of previous studies conflicted about the association between CXCL12 G801A polymorphism and cancer risk. In order to resolve this controversy, the present meta-analysis, which included 4,435 cases and 6,898 controls from 30 case-control studies, explored the association between CXCL12 G801A polymorphism and cancer risk. Our results indicated that CXCL12 G801A polymorphism was associated with an increased risk of cancers.

Additionally, our study contributes the results of subgroup analyses stratified by ethnicity, cancer type and source of controls. Our results indicated that the CXCL12 G801A polymorphism was associated with an increased risk of cancers, especially for breast and lung cancer. However, no significant association was observed for bladder, colorectal and gastric cancers. This is maybe because cancers types differ by carcinogenic mechanisms and environmental exposures and have disparate responses to CXCL12 G801A genotypes. In addition, gene-gene and gene-environment interactions may influence the association between CXCL12 G801A polymorphism and susceptibility to specific cancers [44]–[49]. Furthermore, for some cancer types defined as “other”, only a few studies were published; therefore, it was difficult to detect small, but meaningful associations. Consequently, large-scale and detailed studies are needed to examine these relationships.

In the subgroup analysis by ethnicity, the CXCL12 G801A polymorphism was found to confer an increased cancer risk among Asians under all the genetic models, whereas in the Caucasian subgroup, a significant association was only observed under an additive genetic model and a dominant genetic model. The mechanism that explains this ethnic difference is unknown, but differences in genetic backgrounds and life-styles may contribute to different genetic characteristics and susceptibility to specific cancers. In the present meta-analysis, we failed to find significant relationships between CXCL12 G801A polymorphism and cancer risk in ethnic groups besides Asian and Caucasian. Therefore, more studies in other ethnic groups may be necessary for further progress in this area.

In the subgroup analysis stratified by the source of controls, significant associations were observed in hospital-based studies but not in population-based studies. However, most of the included studies were hospital-based because hospital controls are more readily available. Therefore, the findings in this subgroup should be interpreted with caution. Additional population-based studies are needed to better evaluate this association.

We identified previous genome-wide studies relevant to our research, such as those conducted in breast cancer and lung cancer [50]–[55]. However, these studies were not included in our analysis because their raw data was not available. No significant association between CXCL12 G801A polymorphism and cancer risk was observed in those studies, which conflicts with our results. Possible reasons for this inconsistency are that genome-wide association studies are limited by their relatively small samples and can't contain all kinds of populations.

Two meta-analyses similar to that presented herein were performed by Gong et al. [56] in 2012 and MA et al. [57] in 2012, who also investigated the influence of CXCL12 G801A polymorphism on susceptibility to cancers, with similar conclusions. There were two main differences between these two studies and our study. First, the study of Gong et al. included two articles that deviated from HWE, which were excluded from our study. Second, the literature searches of the two prior meta-analyses were conducted before October 2011 and May 2011, respectively. Since then, several additional studies of the CXCL12 G801A polymorphism and cancer risk were published. Therefore, the sample was larger and the statistical power was greater in our meta-analysis.

We conducted the largest and most comprehensive quantitative meta-analysis of the relationship between CXCL12 G801A polymorphisms and cancer risk. Nevertheless, we recognize some limitations of this meta-analysis. First, our meta-analysis was based primarily on unadjusted ORs with 95% CIs because potential correlative factors, such as environmental factors and other lifestyle habits, were not available. Second, the meta-analysis was limited by the relatively small number of available studies, which limited our ability to perform subgroup analysis for every type of cancer. Third, our analysis was limited to Asian and Caucasian ethnicities, and it is uncertain whether these results are generalizeable to other populations. In addition, cancer is a multi-factorial disease that results from complex interactions between many genetic and environmental factors. Therefore, a single gene or single environmental factor is unlikely to explain cancer susceptibility.

In conclusion, this meta-analysis suggested that CXCL12 G801A polymorphism was associated with an increased risk of cancer based on current published data. In the future, large-scale well-designed studies with more information about potential correlative factors are needed to better estimate possible gene-gene or gene-environment interactions.

## Supporting Information

Checklist S1 **PRISMA 2009 Checklist.**
(DOC)

Checklist S2 **Meta-analysis on Genetic Association Studies Checklist.**
(DOC)
